# Five-Step Forest Bathing Protocol as a Nature-Based Solution for Student Wellbeing in Higher Education: A Research Brief on Insights and Lessons from a Pilot Study

**DOI:** 10.3390/ijerph22040579

**Published:** 2025-04-07

**Authors:** Adriano Bressane, Líliam César de Castro Medeiros, Yasmim Cardoso Damasceno Lima

**Affiliations:** 1 Institute of Science and Technology, São Paulo State University (UNESP), Presidente Dutra Highway, São José dos Campos 12247-014, SP, Brazil; liliam.medeiros@unesp.br (L.C.d.C.M.); yasmim.lima@unesp.br (Y.C.D.L.); 2 Civil and Environmental Engineering Graduate Program, São Paulo State University, Bauru 01049-010, SP, Brazil

**Keywords:** nature solutions, student wellbeing, forest bathing, higher education

## Abstract

*Background*. Students often face high levels of mental distress, which can adversely affect their academic performance and overall wellbeing. While forest bathing, as a nature-based solution (NBS), has recognized benefits for mental wellbeing, its specific impact on undergraduate students remains understudied. *Purpose*. This research brief aims to present the insights and lessons learned from a pilot study utilizing a five-step forest bathing protocol applied to higher education students. *Method*. A semester-long intervention study was conducted in natural urban parks in São José dos Campos, São Paulo, Brazil. Thirty-six newly enrolled university students participated in three NBS sessions, with data collected before and after each intervention using the Depression, Anxiety, and Stress Scale. The interventions were spaced approximately one month apart. For the paired comparison between repeated measurements, one-tailed tests were used based on Student’s *t*-test, with significance set at *p* < 0.05. *Results*: The protocol had significant and substantial effects on reducing anxiety (48.4%, *d_Cohen_* = 0.415), depression (35.4%, *d_Cohen_* = 0.431), and stress (33.5%, *d_Cohen_* = 0.479) in participants, particularly after visiting parks with a higher degree of naturalness. *Insights and Lessons Learned*. The pilot study highlighted the critical role of naturalness in the effectiveness of forest bathing interventions. Parks with more natural elements provided greater reductions in mental distress, supporting theories such as attention restoration theory. Additionally, the findings suggest that integrating NBS programs into university settings can significantly enhance student mental wellbeing and emotional stability. The nuanced responses to different environmental settings underscore the need for well-designed green spaces in academic environments. These insights can inform the design and implementation of green spaces within universities, contributing to improved mental health outcomes for students.

## 1. Introduction

Students often experience high levels of mental distress due to the demands of their coursework, exams, and social pressures, among other factors [1,2,3,4]. Karyotaki et al. [1] examined the association between stress in different areas of life and mental disorders among college students. They found a significant dose–response association and increased odds of having a mental disorder. The study suggests that developing stress prevention interventions could potentially reduce the prevalence of mental disorders among students. Defeyter et al. [2] examined the mental wellbeing of UK university students during the COVID-19 pandemic, focusing on the role of universities. The authors found that a considerable number of students experience low levels of mental wellbeing. Their findings suggest that university leaders should tackle issues such as food and housing security and address mental distress through prevention programs.

These mental health issues can also negatively impact academic performance, increase dropout rates, and hinder relationships and overall wellbeing [5,6,7,8,9,10]. Particularly, first-year engineering students face significant challenges, with some of the highest dropout rates among university programs [11,12,13]. Therefore, finding effective interventions to reduce symptoms of psychological disorders is crucial for promoting the mental health and success of students. One promising approach is forest bathing, a nature-based solution (NBS) which involves immersing oneself in nature and engaging in mindful sensory experiences. While it shares therapeutic goals with broader NBSs, it differs in structure and origin. Nature-based solutions is an umbrella concept promoted by the IUCN and other global frameworks, referring to interventions that address societal challenges through engagement with nature, spanning from ecosystem restoration to urban resilience and human health promotion. In this context, forest bathing can be understood as a subcategory of NBS focused on individual-level wellbeing through experiential, low-impact ecological exposure. The term “forest bathing”, or shinrin-yoku, originated in Japan in the early 1980s as a health-promotion initiative developed by the Japanese Ministry of Agriculture, Forestry, and Fisheries. Rooted in ancient Shinto and Buddhist traditions of nature reverence, shinrin-yoku was institutionalized in Japan as part of preventive medicine and stress reduction strategies, later gaining empirical validation in both physiological and psychological domains.

A growing body of research has established the positive effects of NBSs on overall wellbeing, correlating with diminished stress, elevated mood, and enhanced cognitive function [14,15,16,17]. In agreement, Kim et al. [17] argue that students frequently contend with elevated stress levels and substantial academic pressures, rendering them a susceptible population for mental health challenges. Therefore, investigating the potential of NBSs as an approach to mitigate the stress faced by these students emerges as a promising and viable alternative. Nevertheless, the authors acknowledge that the body of research in this area remains somewhat limited, underscoring the need for further investigation into the efficacy of NBSs for this specific group.

Chiang et al. [18] explored the physiological and psychological responses of individuals to different locations and vegetation densities. The study recruited college students as participants, using a convenience sample of 180 individuals. The findings suggest that the forest interior condition induces stress recovery and is highly preferred, while the forest edge is associated with better attention restoration effects. High-density vegetation improves attentional functioning, but medium vegetation is more preferred. These results can guide designers in selecting optimal site locations and vegetation arrangements for restorative effects.

Kil et al. [19] examined the differences in demographic and visit characteristics, recreation experience preferences, and improved wellbeing outcome preferences based on varying levels of place attachment among practitioners. They found that various on-site recreation benefits and improved wellbeing outcomes were significantly higher among those with higher levels of place attachment. The findings suggest that nature engagement plays a significant role in enhancing positive wellbeing benefits. However, this paper does not focus on the university itself or its students.

The systematic review and meta-analysis by Roberts et al. [16] found a small effect for reduction in depressive mood following short-term exposure to the natural environment. The review did not specifically analyze the effect on depressive mood in students. However, it can be inferred that the findings of the review apply to students as well, since some of the included studies recruited college or university students as participants. In conclusion, the authors noted that the observed effect remains largely unexplained, and the high risk of bias and low quality of studies limit confidence in the results, recommending more studies. Similarly, studies included in the meta-analysis developed by Yao et al. [20] also recruited university students as participants, indicating a relationship between exposure to the natural environment and stress reduction in this population. Nevertheless, the study identified significant heterogeneity between results and a high risk of bias, suggesting that more studies are needed to further understand the relationship between the natural environment and stress in students.

From the aforementioned discussion, the impact of NBSs on academic-related mental distress remains a relatively uncharted field within the realm of research. The existing literature on NBSs primarily comprises studies conducted in non-academic settings or on non-student populations. While these studies provide valuable insights into the broader applications of NBSs, they fail to consider the distinct challenges and opportunities that university environments present. First-year engineering students experience a distinctive blend of stressors, such as academic pressures, social transitions, and financial burdens, which necessitate tailored wellbeing intervention programs [21,22,23].

This research brief aims to present insights and lessons learned from a pilot study utilizing a five-step forest bathing protocol designed to reduce stress, anxiety, and depression symptoms among university students. By exploring the potential benefits for the mental health of this particular academic group, this research brief endeavor aims to lay the groundwork for the development of targeted NBS interventions.

## 2. Literature Review

Forest bathing has been shown to significantly reduce anxiety and improve overall mental health in various populations. A study conducted in the Valdivian temperate rainforest demonstrated that undergraduate students experienced reduced anxiety levels following forest bathing sessions [24]. Similarly, Kotera and Fido [25] found that university students who participated in a shinrin-yoku retreat in Fukushima, Japan, reported increased self-compassion, mindfulness, and a sense of common humanity, indicating broad mental health benefits.

Research consistently highlights the stress-relieving benefits of forest bathing. For instance, an online survey during the COVID-19 pandemic revealed that access to quality greenspaces was crucial for the mental wellbeing of university students, including those with pre-existing mental health issues [26]. This underscores the potential of forest environments to alleviate stress and enhance psychological resilience.

While numerous studies focus on stress and anxiety, fewer specifically address depression. However, the general mental health improvements associated with forest bathing suggest potential benefits for depressive symptoms. The comprehensive environmental exposure involved in forest bathing, which includes natural sounds, sights, and scents, likely contributes to its antidepressant effects.

Despite the recognized benefits of NBSs, there is a notable scarcity of research focusing on university students. Most existing studies either explore general populations or specific non-academic groups, leaving a gap in understanding of the unique stressors faced by students. For example, Slimmen et al. [27] investigated stress-related factors affecting mental wellbeing among university students but did not include forest bathing as a variable. Similarly, Shahadan et al. [28] examined the relationship between physical activity and mental wellbeing among overweight and obese female students, without considering nature-based interventions.

The present study aims to fill this gap by specifically examining the effects of a structured forest bathing protocol on the mental wellbeing of university students. By utilizing the Depression, Anxiety, and Stress Scale (DASS-21) to measure outcomes before and after the intervention, this research provides valuable insights into the applicability and efficacy of forest bathing in academic settings. The findings are expected to inform the design and implementation of green spaces within universities, promoting mental health and academic success among students.

Building upon these foundations, the five-step protocol employed in the present study was informed by traditional shinrin-yoku practices, integrated with mindfulness-based stress reduction (MBSR) principles and adapted to suit university student populations. Each step—ranging from sensory immersion to mindful movement and nutrition—was designed to scaffold attentional restoration and emotional regulation in a progressive manner, reflecting contemporary adaptations of forest therapy for structured interventions [25].

Although forest bathing can be categorized under the broad umbrella of NBSs, it is crucial to acknowledge the considerable heterogeneity within this domain. The term NBS encompasses a wide array of strategies—from ecosystem restoration and green infrastructure to nature-assisted therapies—each with varying degrees of standardization, scientific validation, and cultural origin. In this context, forest bathing occupies a distinct niche: one that merges ecological exposure with intentional sensory and psychological engagement.

The five-step protocol described in this study is not intended as a definitive or universal model of forest bathing, but rather as a structured, context-sensitive adaptation designed for academic settings and student populations. It draws upon core elements of shinrin-yoku but incorporates additional layers such as mindful eating and yoga-based relaxation, which are not traditionally part of Japanese forest bathing practices. As such, it should be viewed as a hybrid intervention, combining experiential knowledge, empirical grounding, and cultural adaptation.

This raises a timely epistemological question: should we seek to differentiate and standardize these diverse nature-based practices, or embrace their contextual pluralism? While taxonomic clarity can enhance replicability and policy alignment, excessive rigidity may stifle innovation and local responsiveness. In line with the psychosocial framework endorsed by the WHO and CRPD, this study embraces a pragmatic approach, prioritizing efficacy, accessibility, and participant resonance over strict definitional boundaries.

## 3. Method

### 3.1. NBS Intervention

Forest bathing involves immersive and contemplative outdoor activities designed to promote physical, mental, and emotional wellbeing. To achieve this, several key aspects must be considered. Ideally, forest bathing should take place in natural settings, though it can be adapted for urban parks with abundant greenery. The presence of trees is essential to ensure an immersive and tranquil natural experience. Central to this NBS is the principle of mindfulness, encouraging participants to be fully present in their natural surroundings while engaging their senses. This necessitates a deliberate reduction in pace, disconnecting from distractions such as electronic devices, and embracing the sensory richness of nature.

### 3.2. Participants

The participants in this study were first-year engineering students from São Paulo State University, a group characterized by high levels of academic pressure and one of the highest dropout rates among university programs [11,12,21]. The participants were to engage in unhurried, deliberate walks through the woods. This leisurely pace would allow them to fully absorb the visual, auditory, olfactory, and tactile dimensions of the natural world. During the practice, the participants were encouraged to (a) listen attentively to nature’s auditory tapestry, which may include birdsong, rustling leaves, and babbling streams; (b) observe visual splendors, such as the interplay of sunlight filtering through trees, the vibrant hues of leaves and flowers, and the intricate shapes of rocks and branches; (c) explore various textures, running their hands along tree bark, the softness of moss, or the refreshing coolness of stream water; and (d) inhale the diverse natural scents, influenced by the array of tree species and plant life.

### 3.3. Protocol

A five-step protocol consisting of nature-based activities was introduced to foster mindfulness, relaxation, and a deeper connection with the natural environment, as outlined in Table 1. The activities in the protocol complement each other to benefit college students: (1) “green mindfulness” helps students cultivate awareness, setting the stage for deeper engagement; (2) “green walking” encourages physical activity and a sense of exploration; (3) “green exercise” further promotes physical health; (4) “green mindful eating” provides an opportunity for relaxation and social connection; and (5) “green yoga nidra” promotes a state of conscious relaxation incorporating nature or environmental elements.

The decision to implement a structured five-step protocol was guided by the need to balance evidence-based therapeutic components with practical feasibility in an academic setting. Each step integrates a distinct dimension of nature-based engagement—mindfulness, physical movement, sensory immersion, nutrition, and relaxation—that aligns with validated constructs in environmental psychology and health promotion literature [25]. The multi-step structure also reflects an intentional design to progressively deepen participants’ connection with nature while fostering emotional regulation. For instance, “green mindfulness” establishes present-moment awareness; “green walking” introduces slow-paced sensory integration; and “yoga nidra” promotes parasympathetic activation through structured relaxation. These elements are rooted in existing shinrin-yoku and ecotherapy frameworks and adapted to university students’ mental health needs and time constraints. In summary, the five-step approach operationalizes an integrative model that leverages complementary modalities to maximize psychological restoration, ensuring both methodological rigor and ecological validity.

### 3.4. Selection of Parks and Assessment of Naturalness

The specific parks chosen for this pilot study were Burle Marx Park, Centro Ambiental Edoardo Bonetti, and Vicentina Aranha Park, selected based on their varying degrees of naturalness. The degree of naturalness was assessed using criteria such as the density and diversity of vegetation, the presence of water features, and the extent of human-made structures. Parks with higher naturalness were characterized by dense, diverse vegetation and minimal built-up areas, whereas parks with lower naturalness had more paved paths and constructed features.

### 3.5. Sampling Process and Participant Selection

The sampling process targeted first-year engineering students who were newly enrolled at São Paulo State University. Recruitment was conducted via email invitations and announcements during orientation sessions. Participants were required to be at least 18 years old and provide informed consent to ensure adherence to ethical guidelines for studies involving human subjects. A schedule was devised for a group of 36 newly enrolled college students who were invited to participate in organized visits to natural urban parks in São José dos Campos, São Paulo State, Brazil (Table 2). Individuals who attended the designated locations and dates were then invited to engage in the NBS interventions. Three interventions were conducted during the academic semester, with approximately one-month intervals between each session.

### 3.6. Statistical Methods

A pilot longitudinal study was conducted, collecting data from 89 repeated measures (*n*) before and after the three interventions: #1 (*n* = 31), #2 (*n* = 27), and #3 (*n* = 31). Participants completed the Depression, Anxiety, and Stress Scale test (DASS-21), a validated tool widely used to assess these psychological constructs. For the paired comparison of pre- and post-intervention measurements, one-tailed Student’s *t*-tests were used. The choice of one-tailed tests was based on the directional hypothesis that the interventions would reduce anxiety, depression, and stress levels. The effect size ($d_{Cohen}$) of the NBS on the DASS was calculated using the following formula:

$$d_{Cohen}=\frac{\mu_{b}-\mu_{a}}{\sqrt{\frac{(n_{b}-1){\sigma ²}_{b}+(n_{a}-1){\sigma ²}_{a}}{n_{b}+n_{a}-2}}}\;\;\;\;\;\;\;\;\;\;\;\;\;\;\;\;\;\;\;\;\;\;\;\;\;\;\;\;\;\;\;\;\;\;\;\;\;\;\;\;\;\;\;\;\;\;\;\;$$

where μ and *σ* are the mean and standard deviation before (b) and after (a) interventions, respectively. According to Funder and Ozer [29], an effect size around 0.2 indicates a small effect, 0.3 a medium effect, 0.4 a large effect, and 0.5 or greater a very large effect size. Analyses were conducted with a one-tailed test power (1 − β) of 0.8, a significance level (α) of 0.05, and a minimum detectable effect size (rho) of 0.4 [30].

### 3.7. Ethical Considerations

The study was approved by the Ethics Committee under approval number 58149622.3.0000.0077. The ethical review ensured adherence to all necessary guidelines, including informing participants about the voluntary nature of their participation and their right to withdraw from the study at any time without consequences.

## 4. Results

**Figure 1:**
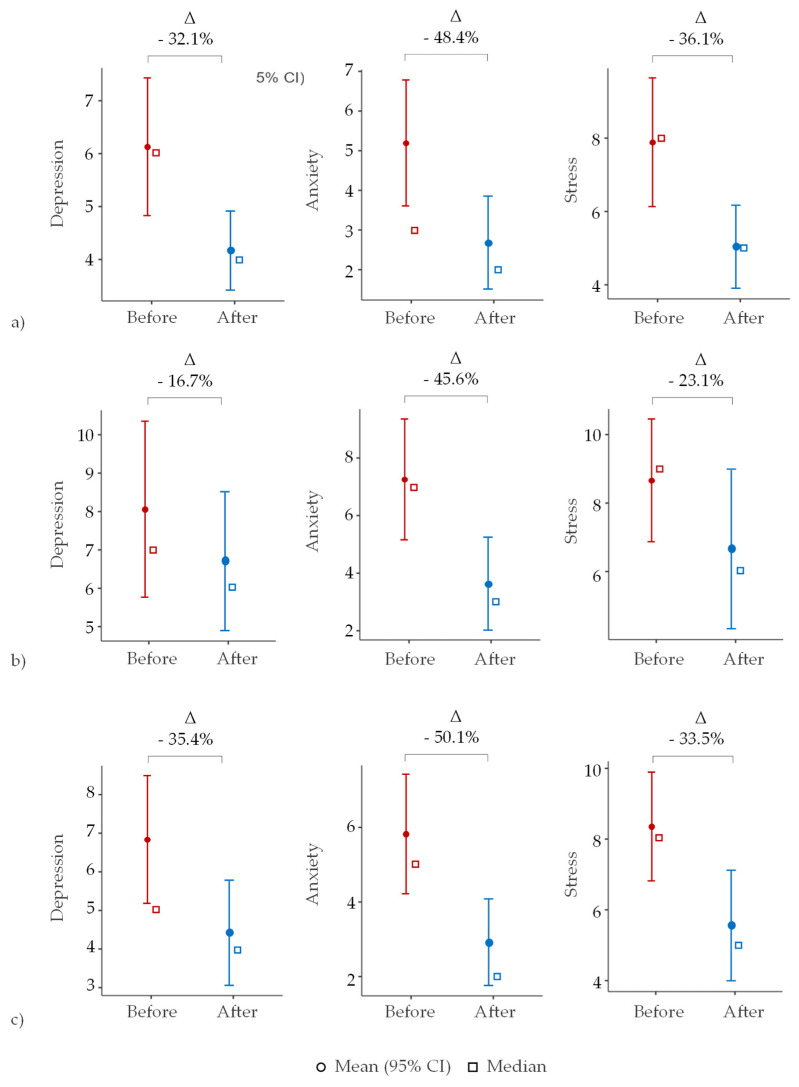
The effect of the forest bathing interventions: (**a**) #1; (**b**) #2; and (**c**) #3.

The impact of forest bathing on students’ levels of depression, anxiety, and stress is summarized in Table 3 and Figure 1, revealing significant improvements across all measured psychological parameters.

Except for depression and anxiety after the second intervention, significant effects (*p* < 0.05) with very large effect sizes ($d_{Cohen}$ > 0.4) were observed in all cases. The greatest reductions in anxiety were noted in interventions conducted in urban parks with a higher degree of naturalness, such as Burle Marx and Edoardo Bonetti. The second intervention at Vicentina Aranha Park, which has a higher proportion of built-up areas, showed less pronounced effects. In addition to the reduction in the average values (μ) of mental wellbeing, the decrease in symptom variability (±σ) suggests greater emotional stability among participants after the interventions.

The findings indicate that the proposed protocol significantly impacts student wellbeing, particularly in reducing anxiety levels and enhancing emotional stability. These significant effects with large effect sizes provide strong evidence for the efficacy of these interventions. However, these findings should be interpreted with caution due to the small sample size, which may limit generalizability and increase the risk of Type I or II errors. In environmental psychology, these results align with the theory of restorative environments, which posits that natural settings can restore cognitive function and reduce mental fatigue, contributing to improved mental distress [31,32]. Supporting this, a systematic review and meta-analysis have shown that exposure to natural environments is associated with reduced negative emotions and fatigue, and improved cognitive functioning and overall satisfaction [33,34,35].

The most significant reductions were observed in anxiety, especially when interventions were conducted in urban parks with a higher degree of naturalness. This outcome supports the attention restoration theory (ART), which suggests that exposure to natural environments can replenish directed attention and reduce anxiety levels. The hypothesis that interventions in more natural settings would yield better results is consistent with this theory [36]. Additional studies have corroborated the theory that green spaces can lower levels of stress, reduce cortisol levels, and improve overall wellbeing, which further validates these findings [37,38].

The differential results between Vicentina Aranha Park (more built-up areas) and Burle Marx Park and Centro Ambiental Edoardo Bonetti Center (higher naturalness) highlight the importance of urban design in enhancing the restorative potential of green spaces. This aligns with the concept of urban green infrastructure, which underscores the role of well-designed green spaces in promoting mental wellbeing in urban settings. These findings are consistent with previous research, such as Chiang et al. [18], which corroborates the theory that areas within forests characterized by high-density vegetation are more effective in promoting stress recovery compared to forest edges.

The decrease in symptom variability after interventions indicates improved psychological resilience, making individuals better equipped to cope with stressors and reducing fluctuations in their mental states. These results align with findings by Kim et al. [19], who observed a reduction in employment-related stress and state anxiety in graduating college students undergoing guided forest therapy.

The significant alleviation of depression and stress after interventions, except in less natural settings, suggests a nuanced interaction between mental distress and natural environments. While anxiety levels appear more immediately responsive to forest-based interventions, symptoms related to depression and stress may require more prolonged or repeated engagement with natural settings to yield comparable benefits. This observation does not imply inherent differences in the nature or pathology of the symptoms themselves, but rather reflects variations in the temporal dynamics of psychosocial recovery processes. Consistent with a psychosocial model of mental wellbeing—as advocated by the World Health Organization and aligned with the CRPD—mental health is understood as a continuum influenced by context, environment, and individual experience. In this light, differences in symptom responses underscore the need for flexible, inclusive intervention designs that accommodate diverse trajectories of recovery. Studies have shown that natural environments can reduce symptoms of anxiety and depression, with the degree of naturalness playing a significant moderating role in the magnitude of these effects [39,40].

The findings emphasize the multifaceted nature of wellbeing. While anxiety, depression, and stress are interconnected, they may respond differently to environmental interventions. Researchers and practitioners should consider this complexity when designing and assessing the impact of interventions aimed at improving mental health. Incorporating NBSs into academic management can improve student retention rates, academic performance, and overall student satisfaction, contributing to a more successful and resilient student body. The significant reductions in anxiety and improvements in emotional stability highlight the importance of providing students with access to natural settings and well-designed green spaces on academic campuses.

## 5. Discussion

This pilot study highlights several important insights and lessons that can be derived from the implementation of a five-step forest bathing protocol aimed at improving the mental wellbeing of university students.

The study demonstrated significant reductions in anxiety, depression, and stress among university students following forest bathing interventions, with a mean reduction in anxiety of 48.4%, depression of 35.4%, and stress of 33.5%. These findings align with previous research indicating the mental health benefits of forest bathing. Langer et al. [24] reported that undergraduate students experienced reduced anxiety levels following forest bathing sessions in the Valdivian temperate rainforest. Similarly, Kotera and Fido [25] found that a shinrin-yoku retreat in Fukushima led to increased self-compassion, mindfulness, and a sense of common humanity among university students, suggesting broader mental health benefits.

The study found that interventions conducted in parks with a higher degree of naturalness had more pronounced effects on reducing mental distress. This supports the ART, which posits that natural environments can replenish directed attention and reduce anxiety levels [36]. Studies by McEwan et al. [41] and Olson et al. [42] have shown that the quality of green spaces, rather than their quantity, is critical for mental wellbeing, particularly among stressed populations such as university students.

The five-step forest bathing protocol was effective in not only reducing symptoms of mental distress but also enhancing emotional stability among participants. This is consistent with findings by Kim et al. [17], who observed reductions in employment-related stress and state anxiety in graduating college students undergoing guided forest therapy. The decrease in symptom variability suggests greater psychological resilience, making students better equipped to handle academic and social stressors.

To translate the study’s findings into actionable strategies, students can autonomously incorporate elements of the five-step forest bathing protocol into their daily routines. For instance, engaging in brief sessions of “green mindfulness” or “green walking” on campus or in nearby green areas may help mitigate academic stress during peak periods. Students with limited access to natural parks can adapt these practices in semi-natural environments such as campus gardens or tree-lined pathways. Furthermore, incorporating short “yoga nidra” or mindful breathing sessions—either independently or through university wellness programs—can reinforce emotional regulation and attentional control. Digital applications that guide mindfulness, sensory grounding, or breathing techniques can facilitate adherence to these practices. These individualized interventions, inspired by the study’s structured protocol, represent scalable, low-cost strategies for cultivating emotional resilience and enhancing mental wellbeing, even outside formal interventions. Promoting student engagement in nature-based self-care practices also aligns with the emerging emphasis on self-directed mental health support within higher education contexts [34,41].

The study’s differential results between more natural and built-up park areas underscore the nuanced relationship between mental distress and natural environments. While significant reductions in anxiety and stress were observed, depression symptoms showed less pronounced improvements in less natural settings. This suggests that while natural environments broadly benefit mental health, specific symptoms may require more tailored or intensive interventions. Bielinis et al. [43] have highlighted that different mental health symptoms respond differently to nature exposure, with greater naturalness being essential for more substantial benefits.

The study emphasizes the potential of integrating NBSs into academic settings to address student mental distress. Incorporating well-designed green spaces on campuses can promote mental health and academic success. This aligns with findings by Bawono et al. [44] and Rodriguez-Redondo et al. [45], who advocate for the inclusion of nature-based therapies in educational institutions to enhance student wellbeing.

One of the key lessons learned is the critical role of green infrastructure in urban design to maximize the restorative potential of green spaces. Educational institutions should prioritize the preservation and creation of natural environments on campuses. The study also suggests that future research should explore the mechanisms by which natural environments promote emotional stability and wellbeing, assess longer or more intensive interventions for various mental health symptoms, and evaluate the broader impact of green infrastructure on mental health within educational contexts.

Recent literature has increasingly characterized Generation Z (individuals born between 1997 and 2012) as a psychologically vulnerable cohort, often referred to as the “fragile generation” due to elevated levels of anxiety, depression, and emotional dysregulation [46,47]. Factors contributing to this profile include digital overexposure, reduced in-person social interaction, academic pressures, and a heightened awareness of global crises. These generational attributes underscore the urgency for tailored wellbeing interventions that resonate with Gen Z’s preferences and needs. Nature-based practices like forest bathing, which emphasize mindfulness, emotional grounding, and sensory engagement, directly counterbalance the overstimulation associated with digital lifestyles. Furthermore, the structure and accessibility of the five-step protocol make it particularly suitable for this generation, who value experiential, flexible, and non-pharmacological approaches to mental health. By aligning intervention strategies with the psychosocial profile of Generation Z, universities can not only promote wellbeing but also foster a culture of emotional resilience and sustainable self-care practices.

Several studies have demonstrated that even brief exposure to natural environments on university campuses can elicit psychological benefits similar to those observed in structured forest bathing protocols. For instance, research by Bratman et al. [37] at Stanford University showed that a 90-min walk in a natural campus setting significantly reduced rumination and neural activity associated with depressive symptoms. Likewise, Richardson et al. [48] have emphasized how simple, everyday activities in nature—such as noticing beauty, engaging emotions, and fostering meaning—are associated with improved wellbeing and pro-nature behaviors. These findings suggest that integrating accessible natural elements into campus infrastructure and daily routines—such as walking paths, contemplative green spaces, and opportunities for mindful engagement—can serve as scalable, low-cost strategies to support student mental health. Rather than replicating formal protocols, universities can draw upon these insights to design context-sensitive interventions that align with their spatial, cultural, and ecological realities.

## 6. Implications for Environmental Psychology

This pilot study provides some preliminary insights for the field of environmental psychology, particularly in understanding how an NBS such as forest bathing can be leveraged to enhance mental wellbeing among university students, as discussed below.

### 6.1. Restorative Environments and Mental Wellbeing

The study supports the ART, which posits that natural environments can restore cognitive functioning and reduce mental fatigue, thereby alleviating stress and anxiety [36]. The significant decrease in anxiety levels (48.4%) and stress (33.5%) in more natural settings aligns with ART’s assertion that immersion in nature replenishes depleted attentional resources, leading to improved mental health outcomes. Environmental psychologists can use these findings to advocate for the integration of natural spaces within academic institutions, promoting designs that maximize exposure to restorative environments [34].

### 6.2. Emotional Stability and Psychological Resilience

The observed decrease in symptom variability suggests that forest bathing not only reduces immediate mental distress but also enhances emotional stability and psychological resilience. This finding is crucial as it indicates that regular exposure to natural environments can help students build resilience against future stressors. Environmental psychologists should consider the long-term benefits of incorporating NBSs into mental health interventions, particularly in high-stress settings such as universities [37].

### 6.3. Design and Implementation of Green Spaces

The differential impacts of parks with varying degrees of naturalness highlight the importance of green space quality in promoting mental wellbeing. Parks with dense vegetation and minimal human-made structures provided greater mental health benefits, suggesting that the design of urban green spaces should prioritize natural elements to maximize their restorative potential. This insight is vital for urban planners and environmental psychologists working on the design and implementation of green spaces in urban areas, especially within educational institutions [39].

### 6.4. Broader Implications

By integrating the principles of environmental psychology with practical interventions like forest bathing, educational institutions can create supportive environments that enhance student mental health and academic performance. This study lays the groundwork for further research into the nuanced relationships between natural environments and mental wellbeing, offering a promising avenue for addressing the mental health crisis in higher education settings.

## 7. Limitations and Future Research

Despite the promising results, this pilot study has limitations that warrant consideration. First, the sample size was relatively small, comprising 36 participants. This limited sample size may affect the generalizability of the findings to a broader population of university students. Future studies should aim to include a larger and more diverse sample to enhance the robustness of the conclusions. Second, the study lacked a control group. Without a control group, isolating the impact of forest bathing interventions on anxiety, depression, and stress levels from changes due to other factors becomes difficult. Future research should incorporate a control group to strengthen the causal inferences that can be drawn from the data. Third, the assessment of the degree of naturalness of the parks was based on qualitative criteria. While this approach provided useful insights, it would be beneficial to develop a more standardized and quantitative method for evaluating the naturalness of green spaces. Such standardization would facilitate comparisons across different studies and contexts. Fourth, the study relied on self-reported measures (DASS-21) to assess mental health outcomes. Self-reported data can be subject to various biases, including social desirability and recall bias. Future research could benefit from incorporating objective measures, such as physiological indicators of stress (e.g., cortisol levels), to complement self-reported data.

Building on the findings and limitations of this pilot study, several avenues for future research are recommended. Future studies should aim to recruit larger and more diverse samples to enhance the generalizability of the findings. Including students from various academic disciplines, as well as different demographic backgrounds, would provide a more comprehensive understanding of the effects of forest bathing on student wellbeing. To establish causal relationships, future research should incorporate control groups that do not participate in forest bathing interventions. This would allow for more definitive conclusions about the efficacy of these interventions in reducing mental distress.

Another avenue for refinement involves accounting for individual personality traits in sample selection and analysis. While this pilot study focused on demographic and academic characteristics, it did not control for or categorize participants based on psychological dispositions such as neuroticism, openness to experience, or trait mindfulness—factors known to influence responsiveness to nature-based interventions [48,49]. Future studies could benefit from incorporating validated personality assessments (e.g., the Big Five Inventory or the Trait Mindfulness Questionnaire) during participant recruitment. Stratifying or matching participants based on such traits may allow for a more nuanced understanding of who benefits most from forest bathing protocols. This approach would also enhance the precision of the NBS as a personalized intervention strategy, aligning with broader trends in individualized mental health care.

Moreover, the temporal structure of the intervention—three sessions distributed across a full academic semester—presents both strengths and limitations. While it ensured repeated engagement with nature, the relatively sparse frequency may not suffice for sustained or cumulative effects, particularly for more persistent symptoms such as depression. Future studies should examine whether increasing the intensity or reducing the interval between sessions yields stronger or more durable outcomes. Additionally, the collective nature of the intervention, even without explicit cooperative tasks, may have introduced social cohesion effects. The shared experience of engaging in forest bathing among peers could itself act as a psychosocial buffer, promoting belongingness and reducing isolation—factors known to influence student wellbeing. Future research should assess the independent and interactive contributions of social participation and nature exposure.

Lastly, it remains unclear how life circumstances between interventions—academic workload, interpersonal stressors, or exposure to other coping mechanisms—may have shaped participants’ psychological trajectories. Incorporating ecological momentary assessments or diary methods in future studies may help disentangle intervention effects from extraneous life variables.

Given the structured nature of the five-step protocol, this study adopted a quantitative, manualized approach to measure psychological outcomes and establish initial efficacy. While forest bathing traditionally emerges from non-standardized, phenomenological roots, the field increasingly requires methodological rigor to compare interventions and inform policy integration. Quantitative designs thus provide a critical entry point for validating therapeutic outcomes, despite their limitations in capturing the full depth of subjective experience. Future studies may complement this approach with qualitative or mixed methods to explore participants’ meaning-making and contextual responses.

Developing standardized, quantitative methods for assessing the naturalness of parks and green spaces would facilitate more precise comparisons across studies. Metrics such as vegetation density, biodiversity, and the presence of water features could be quantified and correlated with mental health outcomes. Incorporating objective measures, such as physiological indicators (e.g., heart rate variability, cortisol levels), alongside self-reported data, would provide a more comprehensive assessment of the impact of forest bathing on mental health. This multimethod approach could help validate self-reported findings and offer deeper insights into the physiological mechanisms underlying the observed effects. Conducting longitudinal studies that follow participants over extended periods would provide valuable information on the long-term effects of forest bathing. Such studies could explore whether the benefits observed in the short term are sustained over time and identify any long-term improvements in mental health and academic performance. Further studies could explore variations in the forest bathing protocol to determine the most effective components and combinations of activities. Comparing different durations, frequencies, and types of nature-based activities could help optimize the design of interventions for maximum benefit.

A broader epistemological reflection emerges from this study and others in the field: is it paradoxical that we must quantify the mental health benefits of nature, which have long been intuitively understood and culturally embedded? While scientific evidence is critical for informing policy and resource allocation, over-reliance on quantification may risk sidelining subjective, existential, and ethical dimensions of human–nature relations. As such, future research should not only diversify its methodological repertoire (e.g., mixed methods, phenomenological inquiry) but also engage with humanistic perspectives that view participants not as objects of measurement, but as meaning-making agents. Recognizing the legitimacy of self-reported experience is essential—not merely as a methodological concession, but as an ethical stance aligned with the values of participatory planning and psychosocial integrity. Repositioning nature access as a right, rather than a utility to be justified, may require us to rethink how evidence is framed and mobilized.

## 8. Conclusions

This research brief introduced a pilot study evaluating a structured forest bathing protocol and demonstrated that even short, periodic interventions can significantly reduce anxiety and enhance emotional stability among college students. Importantly, these outcomes were more pronounced in settings with higher degrees of ecological naturalness, confirming the relevance of environmental quality in nature-based interventions. Key findings include (1) measurable improvements in emotional regulation following exposure to green environments; (2) greater efficacy of interventions conducted in more naturalized settings compared to less natural ones; and (3) indications that stress and depression symptoms may require longer or more intensive exposure to nature for significant change. Based on these results, we recommend that educational institutions (a) preserve and expand high-quality green spaces on campus; (b) integrate low-cost, nature-based interventions such as forest bathing into student wellbeing programs; and (c) adopt policies that recognize green infrastructure as a core component of psychosocial support. This study contributes to the growing evidence base supporting the role of NBSs in mental health promotion, particularly within academic contexts. Future research should further investigate the mechanisms underlying these benefits, assess interventions with higher frequency or duration, and explore the cumulative impact of green infrastructure on student wellbeing, academic retention, and institutional resilience.

## Figures and Tables

**Table 1:** The five-step forest bathing protocol.

Main Steps	Instructions	Time *
Mind-fulness	1.1 Begin with a brief introduction to green mindfulness and its benefits	20
	1.2 Guide participants to find a comfortable spot in nature	
	1.3 Engage in guided meditation, focusing on sensory experiences	
	1.4 Encourage participants to be fully present in the moment	
Green Walking	2.1 Lead a gentle walk through natural environments or nature trails	30
	2.2 Prompt participants to pay attention to shades of green, textures, etc.	
	2.3 Promote mindful walking by slowing down and noticing the surroundings	
	2.4 Allow time for short breaks for participants to touch trees, leaves, etc.	
Green Exercise	3.1 Guide participants through a series of low-intensity exercises in a clearing	20
	3.2 Incorporate grounding techniques, emphasizing contact with the surface	
	3.3 Activities may include balance exercises or simple breathing exercises	
	3.4 Focus on relaxation and connection with natural elements	
Mindful Eating	4.1 Provide participants with a designated picnic area in nature	20
	4.2 Invite them to enjoy a healthy, nature-inspired snack or meal	
	4.3 Emphasize the importance of savoring each bite	
	4.4 Encourage conversation and sharing of experiences during the picnic	
Yoga Nidra	5.1 Set up a comfortable area with yoga mats in a serene part of nature	30
	5.2 Lead a yoga nidra session that integrates natural surroundings	
	5.3 Incorporate poses inspired by nature, such as a tree or mountain pose	
	5.4 Guide participants in deep relaxation and a closing meditation	

* duration in minutes.

**Table 2:** Profile of participants in the NBS.

Intervention	Age	Gender	Total (%)	Cumulative (%)
#1	young adult (18–44 years old)	female	62.8	62.8
		male	35.1	97.9
	middle-age (45–60 years old)	female	1.31	99.2
		male	0.75	100
#2	young adult (18–44 years old)	female	48.8	48.8
		male	40.2	89.0
	middle-age (45–60 years old)	female	6.03	95.0
		male	4.97	100
#3	young adult (18–44 years old)	female	56.7	56.7
		male	38.3	95.0
	middle-age (45–60 years old)	female	2.99	97.9
		male	2.02	100

**Table 3:** The effect of forest bathing on student wellbeing.

NBS		$\mu_{b}$ (±σ)	$\mu_{a}$ (±σ)	**∆** (($\mu_{a}$$-\;\mu_{b}$)/$\mu_{b}$)	p (Shapiro)	p (*t*-Student)	d (Cohen)	Confidence Interval	
								*b*	*a*
#1	depression	6.13 (±3.7)	4.16 (±2.1)	−32.1%	0.142	0.005	0.492	[4.41, 7.45]	[3.40, 4.91]
	anxiety	5.19 (±4.5)	2.68 (±3.4)	−48.4%	0.019	0.014	0.415	[3.58, 6.80]	[1.46, 3.89]
	stress	7.87 (±5.0)	5.03 (±3.2)	−36.1%	0.14	0.008	0.456	[6.08, 9.66]	[3.88, 6.17]
#2	depression	8.06 (±4.9)	6.71 (±3.8)	−16.7%	0.061	0.188	0.221	[6.31, 9.81]	[5.35, 8.07]
	anxiety	7.24 (±4.4)	3.65 (±3.4)	−45.6%	0.777	0.012	0.402	[5.67, 8.81]	[2.43, 4.87]
	stress	8.65 (±4.9)	6.65 (±3.8)	−23.1%	0.959	0.118	0.298	[6.89, 9.90]	[5.29, 8.01]
#3	depression	6.84 (±4.7)	4.42 (±3.9)	−35.4%	0.539	0.011	0.431	[5.16, 8.52]	[3.02, 5.52]
	anxiety	5.81 (±4.5)	2.90 (±3.2)	−50.1%	0.507	0.003	0.532	[4.20, 7.42]	[1.75, 4.04]
	stress	8.35 (±4.4)	5.55 (±4.4)	−33.5%	0.405	0.006	0.479	[6.78, 9.92]	[3.97, 7.12]

*a*—after; *b*—before.

## Data Availability

Due to the nature of this research, participants of this study did not agree for their data to be shared publicly, so supporting data are not available.
